# Three-dimensional fluorescent microscopy via simultaneous illumination and detection at multiple planes

**DOI:** 10.1038/srep31445

**Published:** 2016-08-16

**Authors:** Qian Ma, Bahar Khademhosseinieh, Eric Huang, Haoliang Qian, Malina A. Bakowski, Emily R. Troemel, Zhaowei Liu

**Affiliations:** 1 Department of Electrical and Computer Engineering, University of California, San Diego, 9500 Gilman Drive, La Jolla, California 92093, USA; 2 Department of Physics, University of California, San Diego, 9500 Gilman Drive, La Jolla, California 92093, USA; 3 Division of Biological Sciences, University of California, San Diego, 9500 Gilman Drive, La Jolla, California 92093, USA

## Abstract

The conventional optical microscope is an inherently two-dimensional (2D) imaging tool. The objective lens, eyepiece and image sensor are all designed to capture light emitted from a 2D ‘object plane’. Existing technologies, such as confocal or light sheet fluorescence microscopy have to utilize mechanical scanning, a time-multiplexing process, to capture a 3D image. In this paper, we present a 3D optical microscopy method based upon simultaneously illuminating and detecting multiple focal planes. This is implemented by adding two diffractive optical elements to modify the illumination and detection optics. We demonstrate that the image quality of this technique is comparable to conventional light sheet fluorescent microscopy with the advantage of the simultaneous imaging of multiple axial planes and reduced number of scans required to image the whole sample volume.

The invention of the microscope revolutionized biology, and the capability to see micro-scale biological processes continues to be crucial in both research and industry. The conventional optical microscope is the most common tool for the imaging and monitoring of small structures in a 2D plane. However, many important and interesting biological processes occur in a 3D volume, and conventional microscopy is ill-suited to measure them. In particular, the disciplines of brain imaging^1^,^2^,^3^, nervous system imaging^4^, and neuron activity analysis^5^,^6^ would all highly benefit from 3D microscopy imaging tools.

There are specialized technologies, such as confocal microscopy^7^ and light sheet fluorescent microscopy (LSFM)^8^,^9^,^10^ that can extend the imaging capability into the third dimension. LSFM focuses its illumination and detection at a same plane, minimizes the photo-bleaching from unnecessary excitation and improves image contrast by suppressing out-of-focus emission. By capturing 2D cross-sections at different depths and stacking them along scanning direction, this microscopy modality can be used to record a full 3D map of the sample. Moreover, the recently developed technique of multiple light sheets^11^,^12^ makes the resolution beyond diffraction limit through reduced light sheet thickness and structured illumination method^13^,^14^. However, both confocal microscopy and LSFM techniques typically rely on serial scanning^15^, which is a time-multiplexing technique that limits the imaging speed.

Another approach, multi-focus microscope (MFM)^16^, can record multiple focal planes by using beam splitters to image multiple focal planes on separate cameras. Recently, a new MFM method^17^,^18^ was demonstrated which could image multiple 2D focal stacks simultaneously at one single camera using a single specially designed diffractive element. Thus, multiple measurements at different z plane can be done simultaneously. However, only monitoring multiple planes is not enough to capture clean 3D object information because of the presence of out-of-focus emission. As a result, blurred features and strong background can be observed on each focal plane.

Here, we introduce a novel 3D optical microscopy system named ‘Parallel Multi-Plane Microscopy (PM^2^)’ that selectively and simultaneously illuminates and detects multiple focal planes. PM^2^ implements a scanning array of Bessel beams as illumination source and a multi-focus grating inserted in detection path enabling the imaging of various planes of sample. In addition to the arrangement of hardware, we deployed a de-convolution process of multiple point-spread-functions (PSFs) in order to reduce the ‘crosstalk’ between multiple focal planes. We utilize the proposed 3D microscopy platform for both fluorescent micro-beads and biological samples (*Caenorhabditis elegans*) and show that several object planes can be simultaneously monitored, with remarkably improved quality that is close to ‘light sheet’ microscopy.

## Results

### Simultaneous illumination and detection of multiple planes

**Figure 1:**
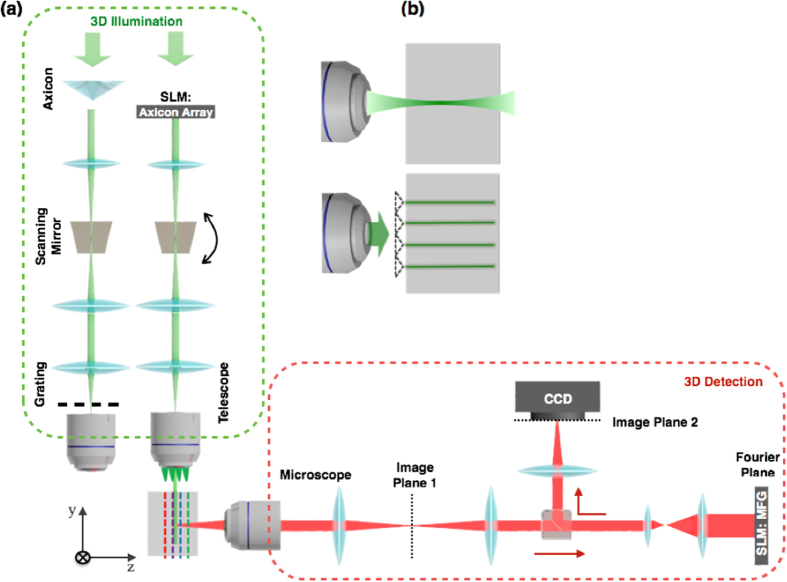
System schematics. (**a**) The optical set-up. The illumination light (shown in green) comes from a collimated laser source and is modified to generate multiple Bessel beams. An array of Bessel beams can be generated either be by an SLM (Right) or by a single axicon that forms one Bessel beam which then can be split using a 1D grating located at the rear pupil of illumination objective lens (Left). Multiple planes (depicted by red, purple, blue, and green dashed lines) are illuminated by an array of Bessel beams scanning along × direction. The detection light (shown in red) collected by a microscope system, is modified by MFG grating pattern on another SLM, before it forms an image on CCD. (**b**) Schematics of multiple Bessel beam illumination. Unlike conventional light sheet illumination (left), the illumination arm (right) has multiple (four is shown here) Bessel beams hitting sample simultaneously.

Our system, which we refer to the PM^2^, consists of two main units: (i) the illumination arm and (ii) the detection arm. A diagram of the experimental implementation of the PM^2^ is shown in Fig. 1a. To simultaneously illuminate multiple isolated planes (x-y planes) in the sample, the illumination arm employs an array of scanning Bessel beams. Instead of Gaussian beams that are commonly used, Bessel beams are used because of their ‘diffraction-free’ property which makes them well confined during propagation (Fig. 1b). The array of Bessel beams are generated by either a liquid crystal spatial light modulator (LC-SLM or SLM) (right) or an axicon combined with a diffractive grating (left): the LC-SLM is designed to directly mimic an array of axicons that generate multiple Bessel beams; and the Bessel beam generated by a real axicon is split into multiple Bessel beams by passing through the 1D grating inserted at the rear pupil of the illumination objective lens (IO) (Zeiss, 10X/0.3). A 1D galvo-mirror is used to scan the resulting Bessel beam array along x-direction.

For the detection arm, which is perpendicular to the illumination arm, we apply a diffractive element, multi-focus grating (MFG)^18^, in addition to the wide-field fluorescence microscope system. With the MFG located at the conjugated Fourier plane of the detection objective lens (DO) (Olympus, 10X/0.25), light collected by detection microscope system is split into multiple paths with equal energy distribution, so the split light beams are directed to different regions on the image sensor (Andor CCD, iXon 897). Meanwhile, the MFG generates diffraction-order-dependent spatial phase modulation that enables the light in different light paths represent different focal planes.

**Figure 2:**
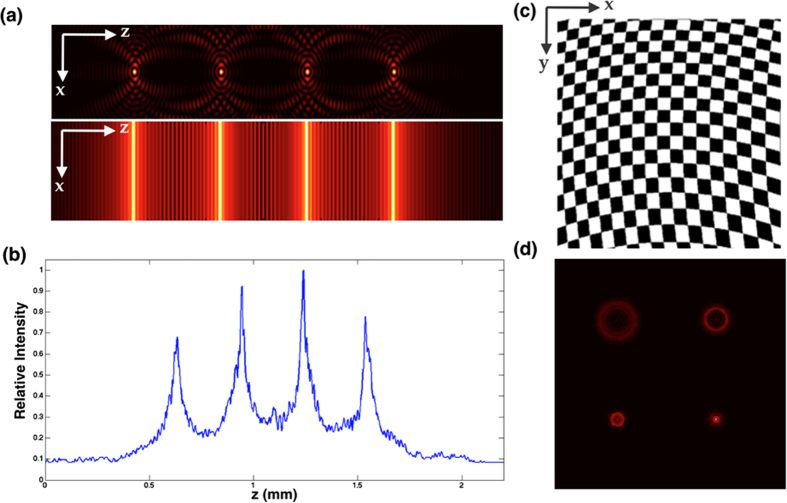
Illumination and detection at multiple planes. (**a**) Bessel Beam intensity profile, where four planes illumination is generated by an array of axicons implemented by spatial light modulator. Top image: four static Bessel beams at sample; Bottom image: time-averaged intensity by sweeping beams along × direction. (**b**) Measured Cross-section of time averaged illumination profile. Four focal planes have adjacent separation 300 μm. (**c**) Schematics of multi-focus grating (MFG), which can monitor four planes. MFG is a binary phase grating, white indicates 0 phase shift, black indicates half a period (π) phase shift. (**d**) Example of a point source imaged through the MFG. Point source only focus at one desired diffraction order (right bottom).

An example of four plane illumination and detection is shown to demonstrate the feasibility of our prototype. Four illumination planes, as depicted in Fig. 2(a), are generated by spatial light modulator, which has a phase modulation pattern of an array of four axicons. Its time-averaged illumination profile, as depicted in Fig. 2b, is measured by a CMOS camera (Thorlabs, DC1645) with long time exposure (>1s) and Bessel beam scanning period 5 ms. The thickness (FWHM) of illumination plane is 30 μm with 300 μm separation between adjacent planes. FWHM is determined by both the angle of axicon and the demagnification caused by illumination objective (IO) lens. It is important to note that FWHM of the illumination plane can be significantly reduced to the scales close to diffraction limit by optimizing magnification of IO lens and design of grating patterns at the rear pupil plane^12^. In order to match the number and separation of multiple illumination planes, a binary multi-focus grating (MFG) is designed (Fig. 2c). The pattern of the MFG grating unit defines the energy distribution into multiple diffraction orders, while the geometrical distortion of the grating pattern, as can be observed in Fig. 2(c), generates required phase modulation.

This diffraction order dependent phase modulation, which can also be referred as defocus ability, can be understood by imaging a point source through MFG. Compared to conventional gratings, which have no extra spatial phase modulation and all diffraction orders are identical, MFG modulates input wave and makes different diffraction orders focused at different z locations. As depicted in Fig. 2d, only one out of four diffraction orders focuses the point source at a given z plane, while other diffraction orders form blurred images defined by defocused point spread function (DPSF) of imaging system.

### Multi-PSFs Deconvolution

**Figure 3:**
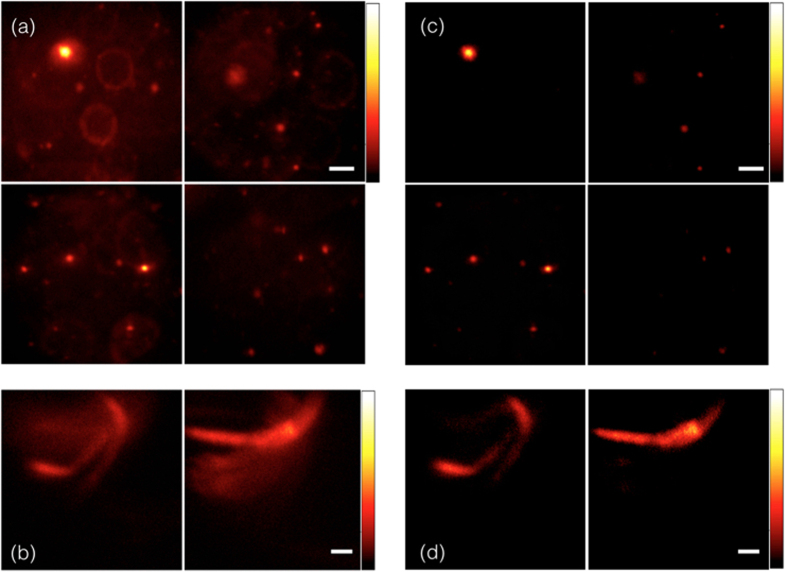
Multi-PSF deconvolution. (**a**,**b**) Raw Data. (**a**) Four focal planes captured by a single camera frame. Each two adjacent planes are separated by 300 μm. (**b**) Two focal planes of one sample which are 200 μm apart captured by a single camera shot. Reconstruction results after applying four-PSF deconvolution process in (**c**) and two-PSF deconvolution process in (**d**). Sample: (**a**,**c**) Randomly distributed 10 μm & 500 nm fluorescent beads (540/560 nm) in PDMS. (**b**,**d**) *C*. *elegans* (GFP labeled). Scale Bar: 40 μm. Objective lens: 10X/0.25.

The PM^2^ set-up is capable of acquiring information from multiple focal planes while out-of-focus information is suppressed. However, on each focal plane, information from other focal planes form blurred features and increase the background, referred as ‘crosstalk’ between multiple focal planes (Fig. 3a,b). Since limited number of focal planes get illuminated and detected, one can estimate the ‘crosstalk’ between those planes by a series of defocused point spread functions (PSF). As a result, a de-convolution algorithm can considerably reduce this ‘crosstalk’ problem. A large-scale l1-regularied least square (*l1_LS*) algorithm^19^ is applied to invert the cross talking impact. In order to experimentally verify our imaging system and image processing algorithm, a mixture of randomly distributed fluorescent beads are imaged by PM^2^. Imaged sample is a mixture of 10 μm and 500 nm 540/560 nm fluorescent beads from Invitrogen Corporation fixed in PDMS (product no. Sylgard 184 Silicone Elastomer) from Dow Corning Corporation. A 10 mW 532 nm laser is used as excitation source and a 560 nm detection filter with 10 nm bandwidth is used. Array of Bessel beams scan along lateral (x) direction to form multiple effective illumination planes, with various planes separation. Figure 3(a) shows four focal planes captured by a single camera shot. Each two adjacent planes are separated by 300 μm. ‘Crosstalk’ between multiple focal planes causes ring-like structure in the raw data and also downgrades the image contrast. Figure 3(c) shows same four focal planes after the multi-PSFs de-convolution and only focused information lefts at each focal plane.

The same de-convolution method was applied to a sample of *C*. *elegans* nematodes, with two focal planes imaged simultaneously (deploying two estimated PSFs). This figure clearly shows that that the final reconstructed image (Fig. 2d) is well in focus, although in-focus and out-of-focus emission are overlaid in the raw data (Fig. 2b). Therefore, the ‘crosstalk’ impact was digitally minimized so that overall performance (resolution and contrast) of our multi-frame imaging system is improved drastically.

**Figure 4:**
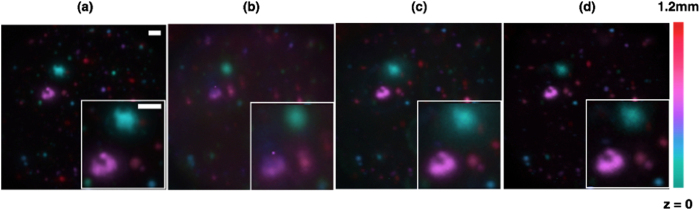
(**a**) LSFM: Light sheet fluorescent microscopy with 60 scanning frames. (**b**) MFM: Multi-focus microscopy with 15 scanning frames. (**c**) PM^2^ Raw: Multi-focus plus multiple light sheet illumination microscopy with 15 scanning frames. (**d**) PM^2^ Deconvolution: Final Result after post-processing PM^2^ raw data with de-convolution algorithm. Sample: Fluorescent beads (540/560 nm) randomly distributed in PDMS. Sample Volume: 290 μm × 290 μm × 1200 μm. Detection Objective Lens: Olympus 10X/0.25. Scale Bar: 20 μm.

To further demonstrate the performance of PM^2^ set-up, a comparison among light sheet fluorescent microscopy (LSFM), multi-focus microscopy (MFM) and our parallel multi-plane microscopy (PM^2^) was made. The configuration of four focal planes with 300 μm separation was used. A volume of fluorescent beads is imaged through scanning the sample stage along z direction with a 20 μm step. It takes 15 scans in order to acquire entire 3D volume. For comparison, Fig. 4(a) shows a LSFM image taken by 60 camera frames to cover the same z range. All 3D volume data are then overlaid in 2D, with ice color map indicating focal depth information. Figure 4(c) shows a raw data captured by PM^2^ system, which is better contrasted than MFM (Fig. 4b), but worse than LSFM (Fig. 4a). This can be understood that PM^2^ system suppresses out-of-focus emission but remain suffering from unnecessary emission from other in-focus planes. By applying multi-PSFs de-convolution algorithm, emission from other focal planes gets substantially depressed and reconstructed data (Fig. 4d) now is close to LSFM data. It should be noted that although the algorithm helps reduce the crosstalk between multiple planes and makes reconstructed data higher contrasted, the absolute illumination power at each focal plane is less due to split of energy. Lower illumination power will also result in lower signal to noise ratio, which is the ultimate limit of multi-focus technique and cannot be improved by this algorithm.

As expected, the introduction of the MFG shouldn’t drastically affect the optical resolution, so that the whole PM^2^ system is close to diffraction limited (or NA limited). By using a single 200 nm fluorescent bead as a test, a regular fluorescent microscope shows FWHM ~1.27 μm under a 10X/0.3 objective lens. The PM^2^ with the same objective lens achieved resolution ~1.32 μm (Supplemental Fig. 1). Slight image broadening along the grating diffraction direction was also observed, but can be solved by adding a dispersion compensation element^18^. Given that the pixel size of the CCD is fixed, the image resolution (~3.5 μm as shown in Supplemental Fig. 2) is traded for a larger field of view in the demonstration of parallel imaging capabilities shown in Figs 3 and 4. A larger format of sensor CCD camera with smaller pixel size is always preferred, so that neither of the field of view nor the resolution has to be sacrificed.

### 3D Imaging of a volume of *C*. *elegans*

**Figure 5:**
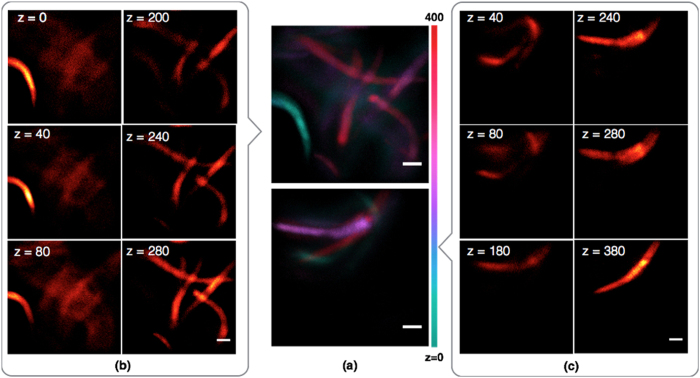
Imaging results of a 3D volume of *C*. *elegans* after deconvolution. PM^2^ system has two focal planes (z planes) illuminated and detected simultaneously. Two focal plane has separation 200 um. Lateral field of view per focal plane: 340 μm × 340 μm. Objective lens: Olympus 10X/0.25. Sample is scanned along z direction for 10 times, with a scanning step 20 μm. (**a**) presents 3D information as a stack of focal planes. Different colors represent different focal plane along ‘z’ direction. Top and bottom figures in (**a**) are two independent measurements at different location of the *C*. *elegans* sample. (**b**,**c**) A few selected 2D focal planes from (**a**). Each two focal planes (separation: 200 μm) at same row are simultaneously imaged by a single camera. Sample: randomly distributed *C*. *elegans*. Sample density: 16000 animals/100 uL. Scale Bar: 40 μm.

In order to demonstrate the capability of PM^2^ for bio imaging, we imaged *C*. *elegans* larvae expressing GFP in the intestine and hypodermis. Investigating *C*. *elegans* has drawn considerable attention in science, since it can be deployed as a model for genes and neural systems in animals with more complex structures^1^,^20^. We conducted imaging experiments with our proposed 3D microscopy system on a volume of worms placed inside a glass capillary. Figure 5 depicts multiple *C*. *elegans* samples in a small volume (0.35 mm × 0.35 mm × 0.40 mm). Two focal planes separated by 200 μm are imaged simultaneously on CCD by designed multi-focus grating. Then sample is scanned along z-axis, with a step of 20 μm. It takes 10 scans to cover entire 400 μm range. Figure 5(b1,b2) shows a few selected focal planes, while (a1,a2) presents a stack of 20 focal planes with hot colormap representing z dimension. All images are processed by multi-PSF deconvolution algorithm to minimize ‘cross-talk’. As can be seen in this figure, all samples have been imaged and their spacing from each other is quantified, suggesting the application of fast cytometry or particle counting of the proposed platform with microscopy imaging quality.

## Conclusion

While conventional light sheet fluorescent microscopy provides high contrast 3D images of biological samples, the necessity of having a high speed imaging that can bring a complete map of inner structures of fluorescent samples still exists. PM^2^ is a technique that combines multiple existing technologies to exploit the possibility of using a 2D conventional microscope to capture clean 3D information with high speed. It only illuminates and detects those fluorophores that are in focus. Thus, it provides multiple focused images with high contrast and better resolution as result of SNR enhancement.

Both lateral resolution and axial resolution of this prototype system are DO lens (NA) limited, since light sheet thickness is larger than the depth of field of objective lens (10 μm for a 10X/0.25 Olympus). Consequently, this imaging platform has more potential to take full advantages of multiple light sheets. Future development is suggested to focus in two directions: 1) To implement super-resolution technology by creating thinner light sheets and utilizing structured illumination method; 2) implement under-sampling to get reconstructed data from less measurement or lower light level. In addition, though Liquid Crystal Spatial Light Modulator (LC-SLM) is used in our prototype set-up, due to its high flexibility of switching modulation patterns, it has low transmission/reflection efficiency and can be replaced by a series of fabricated phase grating to maximize light collecting efficiency.

It should be noted that the reason we chose to work with up to four scanning Bessel beams (and thus, imaging four parallel planes concurrently) is the trade-off between number of focal planes and light intensity on each focal plane. Simultaneous imaging more focal planes results in a light intensity reduction at on each focal plane, and thus, it degrades the signal to noise ratio (SNR) and noise limited resolution. Moreover, increasing the number of imaging planes reduce the effective area each plane images and projects on CCD plane, since the CCD frame area is constant and captured frames of parallel frames cannot overlap. The latter drawback can be overcome by switching to an imaging sensor with larger image sensor area; however, the first problem is the ultimate limit of all multiple-focus microscopy technologies. By choosing suitable number of focal planes and plane separations based on sample’s lighting condition, the introduced PM^2^ system can be deployed for live monitoring of biological samples and intracellular activities (e.g. monitoring neural activity).

## Methods

### Bessel Beam Generation by a spatial light modulator

The electric field profile to create a Bessel beam can be described as^21^


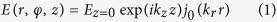


where *r* denotes the radial dimension, Φ is used for angular shift, and *k*_*z*_ and *k*_*r*_ represent wave vectors in axial and radial directions. As an immediate result, the intensity profile obtained by the mentioned electric field distribution is proportional to 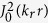. Spatial phase modulation pattern of an axicon can be written as:


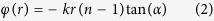


where *α* is the wedge angle of axicon, *n* is refractive index of axicon, 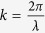 where *λ* is the laser light wavelength. A practical approximation to implement the phase profile of axicon is the binary form which is the digitized format of normal axicon with only two phase levels (*0* and π) along the radial direction.

### Multi-focus grating pattern design

The relationship between MFG geometrical distortion and phase modulation can be represented by Eq(3).


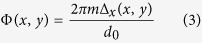


where Φ is spatial dependent phase changes, Δ_x_ is the × displacement of the grating pattern from its undistorted position, d_0_ is grating period before distortion, and *m* is the diffraction order^9^,^10^.

In order to capture images from multiple focal planes with separation z, the MFG is designed based on Eq (4).


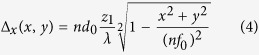


where z_1_ is the distance between actual focal plane and default focal plane of objective lens, *n* is refractive index of sample media, d_0_ is grating period before distortion, *λ* is working wavelength, and *f*_0_ is the effective focal length of the objective lens. The Abbe sine condition is considered in Eq (4) to correct spherical aberration. 2D geometrical distortion (Δ_*x*_ and Δ_*y*_) needs to be considered to get four focal planes.

### Multiple PSF De-convolution

Four focal planes, I_1_, I_2_, I_3_, and I_4_ are detected by a single snapshot of one image sensor array. Original objects distribution at each focal plane is represented by O_1_, O_2_, O_3_ and O_4_. The image formed at each focal plane is an incoherent super position of the focused image corresponding to the objects in that particular plane as well as the blurred images of neighbor planes. If we define the point spread function (*PSF*) when the object and image are in the same focal plane as PSF_1_, the blurring PSF from the impact of the object plane in the most adjacent plane as PSF_2_, and of the farther adjacent plane as PSF_3_ and PSF_4_. We can obtain the relation between what is captured by the image sensor and object distribution at each focal plane as following:


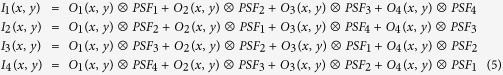


PSFs are estimated to be Gaussian, based on numerical aperture (*NA*) of objective lens and separation of focal planes.

Above equations are then further organized in order to convert the matrix convolution operator to matrix multiplication, so that the intensity distribution captured by image sensor would have the following relation with the object distribution to be calculated.


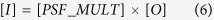


where [*I*] represents the vectorized form of the intensity distribution at each image sensor frame, [*PSF*_*MULT*] is the Teoplitz matrix derived from all PSF distributions, and [*O*] is the un-blurred (focused) object distribution at image sensor frame which is to be calculated by solving the mentioned equation. l1-LS (l1 regularized least square) algorithm^19^ is deployed to solve this problem, which has been shown to be fast and noise-robust when being used for *PSF* deconvolution^22^,^23^.

### *C*. *elegans* sample preparation

*C*. *elegans* strain ERT10 (*jyIs2*) [*pmp-5::GFP*] was made with the following steps. The *pmp-5* promoter-GFP fusion was made with overlap PCR using 2.5-kb upstream of *pmp-5* placed in front of GFP. This fusion product was injected into N2 animals, along with a *myo-2:: mCherry* coinjection marker that expresses in the nuclei of pharyngeal muscle cells. One of the extrachromosomal array lines was integrated using UV- irradiation to generate the integrated transgene *jyIs2*, which expresses GFP in the intestine and epidermis.

*C*. *elegans* strain ERT010 *jyIs2*[*pmp-5::GFP*] was maintained at 20 **°**C on nematode growth media (NGM) and fed with *E*. *coli* strain OP50, as described^24^. Synchronized ERT010 *jyIs*2[*pmp-5::GFP*] L1s were grown for 24 hours, then fixed in 4% PFA for 15 min, and embedded in 1% agarose, which was introduced into a glass capillary containing 16000 worms/100 μL of agarose.

## Additional Information

**How to cite this article**: Ma, Q. *et al*. Three-dimensional fluorescent microscopy via simultaneous illumination and detection at multiple planes. *Sci. Rep.* **6**, 31445; doi: 10.1038/srep31445 (2016).

## Supplementary Material

Supplementary Information
