# Supplementary material for: Three-dimensional fluorescent microscopy via simultaneous illumination and detection at multiple planes
Source: Sci Rep. 2016 Aug 16;6:31445. doi: 10.1038/srep31445 (PMC4985622; doi:10.1038/srep31445)
Supplement: Supplementary Information [file srep31445-s1.pdf]

Three-dimensional fluorescent microscopy via simultaneous illumination and detection at multiple planes

Qian Ma[1], Bahar Khademhosseinieh[1], Eric Huang[2], Haoliang Qian[1], Malina A. Bakowski[3], Emily R. Troemel[3], and Zhaowei Liu[1,†]

[1]Department of Electrical and Computer Engineering, University of California, San Diego,

[2]Department of Physics, University of California, San Diego

[3]Division of Biological Sciences, University of California, San Diego,

9500 Gilman Drive, La Jolla, California 92093, USA

[†]Corresponding author email address: zhaowei@ucsd.edu

Supplementary Figures

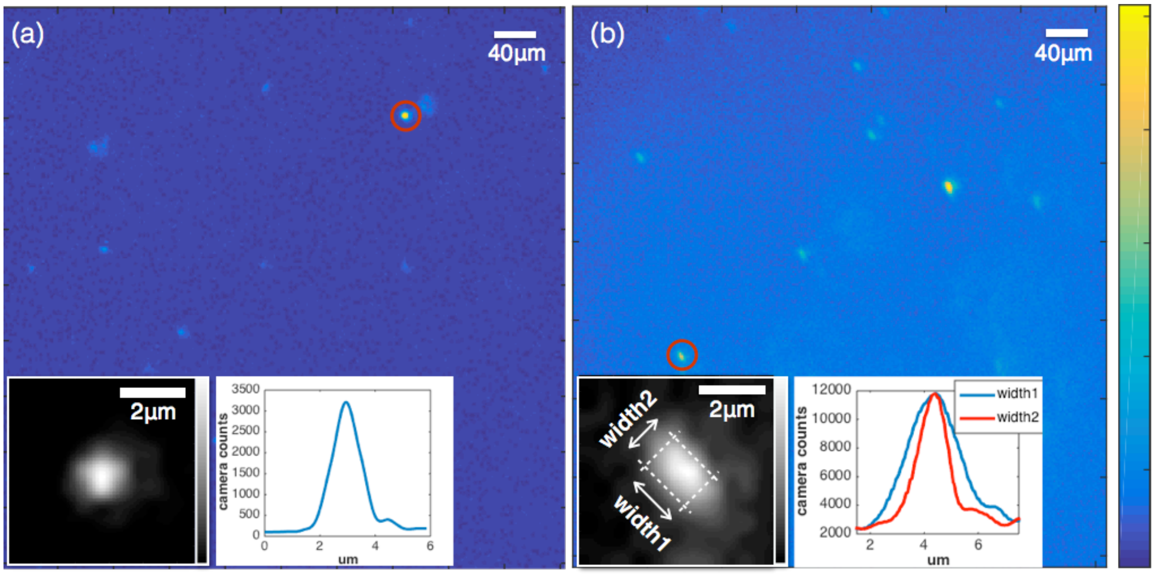

Supplementary Figure 1. Image with NA limited resolution. Imaged sample: 200nm fluorescent beads sparsely distributed in Agar. Pixel size at sample plane: 0.528 µm. Objective lens: Zeiss, 10X/0.3. (a) Camera Image, zoom in image and its cross section without MFG. Both zoom in image and its cross-section are interpolated. Measured FWHM: 1.27 µm. (b) Camera Image, zoom in image and its cross section with MFG. Both zoom in image and its cross-section are interpolated. Measured FWHM: Width 1 is 2.22 µm. Width 2 is 1.32 µm.

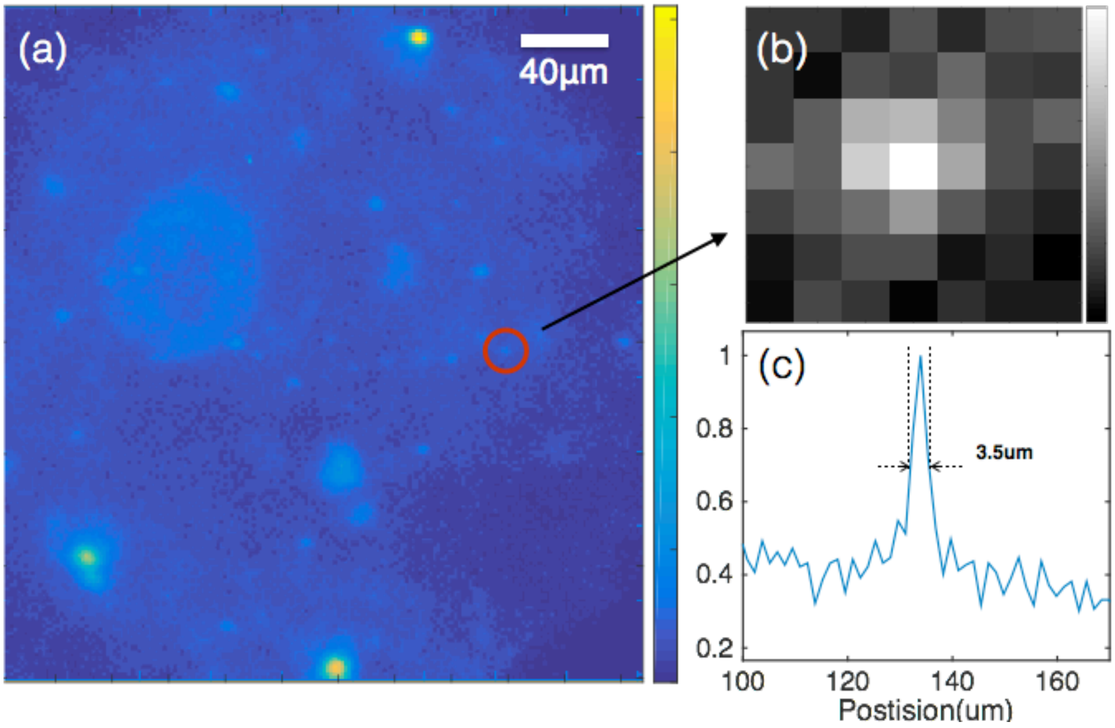

Supplementary Figure 2. Image with Camera pixel size limited resolution. (a) Raw image from the same data set as figure 3 and figure 4 in the manuscripts. Pixel size at sample plane: 1.44 μm; Imaged sample: Mixed 10 μm and 500 nm fluorescent beads in PDMS. (b) Magnified image of a single bead. (c) Cross section of a single bead image.
